# Effect of aprepitant on kynurenine to tryptophan ratio in cART treated and cART naïve adults living with HIV

**DOI:** 10.1097/MD.0000000000025313

**Published:** 2021-06-11

**Authors:** Sergei Spitsin, Vasiliki Pappa, Annemarie Kinder, Dwight L. Evans, Jay Rappaport, Steven D. Douglas

**Affiliations:** a Children's Hospital of Philadelphia Research Institute; b Perelman School of Medicine, University of Pennsylvania, Philadelphia, PA; c Tulane National Primate Research Center, Covington, LA, USA.

**Keywords:** aprepitant, HIV, kynurenine, neurokinin-1, substance P, tachykinins, tryptophan

## Abstract

Changes in tryptophan metabolism affect human physiology including the immune system, mood, and sleep and are associated with human immunodeficiency virus (HIV) pathogenesis. This study investigates whether the treatment of HIV-infected individuals with the neurokinin-1 receptor antagonist, aprepitant, alters tryptophan metabolism.

This study utilized archival samples from 3 phase 1B clinical trials “Anti-HIV Neuroimmunomodulatory Therapy with Neurokinin-1 Antagonist Aprepitant”-2 double-blinded, placebo-controlled, and 1 open-label study. We tested samples from a total of 57 individuals: 26 combination antiretroviral therapy (cART) naïve individuals receiving aprepitant, 19 cART naïve individuals receiving placebo, and 12 individuals on a ritonavir-containing cART regimen receiving aprepitant. We evaluated the effect of aprepitant on tryptophan metabolism by measuring levels of kynurenine and tryptophan in archival plasma samples and calculating the kynurenine to tryptophan ratio.

Aprepitant treatment affected tryptophan metabolism in both cART treated and cART naïve individuals with more profound effects in patients receiving cART. While aprepitant treatment affected tryptophan metabolism in all HIV-infected patients, it only significantly decreased kynurenine to tryptophan ratio in cART treated individuals. Aprepitant treatment offers an opportunity to target inflammation and mood disorders frequently co-existing in chronic HIV infection.

## 1 Introduction

Human immunodeficiency virus (HIV) infection is associated with conditions linked to chronic inflammation and increased tryptophan catabolism, leading to increased kynurenine, the downstream metabolite, which is involved in the control of immune responses and inflammation. The activity of indoleamine 2′3′-dioxygenase (IDO), the rate-limiting step in the kynurenine pathway, is determined by the kynurenine to tryptophan ratio (KTR), with increased KTR representing increased IDO activity. IDO activation is linked to immune suppression/immune activation during HIV infection.^[1]^ Several studies demonstrated increased KTR in HIV-infected individuals and a correlation between increased KTR and progression to acquired immune deficiency syndrome (AIDS).^[2–5]^ IDO upregulation may deplete tryptophan, which is a precursor for melatonin and serotonin. Additionally, IDO can metabolize melatonin and serotonin directly as a substrate, in the absence of alterations in tryptophan levels.^[6]^

Serotonin and its receptors activate pathways that are implicated in mood regulation and are pharmacologic targets in depression treatment.^[7,8]^ Stress and depression are implicated as risk factors in the morbidity and mortality of a wide range of human diseases including, HIV, cancer, cardiovascular diseases, and diabetes,^[9]^ and they may impair key components of cell-mediated immunity,^[10–14]^ as well as heighten susceptibility to infectious diseases,^[15–17]^ including HIV infection^[9,18–27]^ both before and after the advent of combination antiretroviral therapy (cART).^[25]^ The specific immune mechanisms by which stress and depression may influence immunity and HIV disease progression and mortality are not fully understood.

Several studies documented the widespread distribution of serotonin receptors and transporters on monocytes, macrophages, T-cells, and natural killer (NK) cells (reviewed^[1]^). Serotonin modulates NK and T-cell function by enhancing the cytolytic activity of NK cells, possibly through the activation of serotonin receptors on monocytes^[28,29]^ and T-cells.^[30]^ Our ex vivo studies extend these data and suggest that selective serotonin reuptake inhibitors restore immune functions by directly decreasing inflammation and increasing NK cytolytic activity.^[31–33]^

In a series of clinical phase 1B trials “Anti-HIV Neuroimmunomodulatory Therapy with Neurokinin-1 Antagonist Aprepitant,” we targeted chronic inflammation in HIV infection.^[34–36]^ Details of aprepitant administration protocols are listed in Table 1. Aprepitant treatment had no effect on viral load in both cART naïve and cART treated patients.^[34–36]^ Aprepitant treatment had an overall anti-inflammatory effect in HIV-infected individuals, although the pattern of the affected molecules was different between cART treated and cART naïve individuals.^[34,35]^ Aprepitant treatment decreased plasma levels of the neurokinin-1 receptor ligand, substance P (SP), and IL-6 in cART treated and naïve individuals, but in cART naïve individuals it also decreased the levels of additional pro-inflammatory markers, such as TNFα, MIP-1α, G-CSF, IL-8, sCD163, and PD-1 in CD4+ T-cells^[35]^ (for additional information on changes in individual markers of inflammation see supplemental material to Tebas et al^[35]^). In cART treated individuals, an analysis of plasma levels of 1300 proteins using the SOMAscan assay identified 176 proteins and several metabolic pathways that were affected by aprepitant treatment including inflammation, immune response, apoptosis, cell adhesion, and lipid metabolism^[34]^ (for the complete list of markers affected by aprepitant treatment as well as changes in viral load and metabolic profiles see supplemental material to Spitsin et al^[34]^).

**Table 1:** Summary of patient populations and aprepitant administration protocols.

Study description	Study objectives	Patients and treatment	Study duration
Study #1	Primary objectives: to evaluate the safety and tolerability of 2 different doses of aprepitant in cART naïve HIV-infected individuals and to assess the response of plasma HIV-1 RNA	9 subjects received 125 mg of aprepitant	2 weeks of aprepitant treatment followed by off drug for additional 4 weeks
Phase IB randomized, placebo-controlled, double-blinded study	Secondary objectives: to evaluate the dose-response and pharmacokinetic and pharmacodynamic relationship between viral RNA change and aprepitant plasma levels, the effects on CD4+ and CD8+ T-cell counts and circulating SP levels	8 subjects received 250 mg of aprepitant daily	
(see ref 35 for details)		10 subjects received placebo	
Study #2	Primary objectives: to evaluate the safety, and tolerability of 375 mg of aprepitant daily in cART naïve HIV-infected individuals and to assess the response of plasma HIV-1 RNA	9 subjects received 375 mg of aprepitant per day	2 weeks of aprepitant treatment followed by off drug for additional 4 weeks
Phase IB randomized, placebo-controlled, double-blinded study	Secondary objectives: to evaluate immunomodulatory and anti-inflammatory properties of aprepitant and aprepitant pharmacokinetics	9 subjects 375 mg of aprepitant per day	
(see ref 34 for details)		9 subjects received placebo	
			
Study #3	Primary objectives: to assess the safety and tolerability of aprepitant when administered in combination with a ritonavir-containing antiretroviral regimen; to assess the pharmacokinetic characteristics of aprepitant when co-administered with cART; to evaluate the effects of aprepitant on plasma levels of SP and sCD163	12 subjects received 375 mg of aprepitant per day	4 weeks of aprepitant treatment followed by off drug for additional 4 weeks
Phase 1B open-label study	Secondary objectives: to evaluate the effects of aprepitant on levels of CD4/PD-1; to evaluate the effects of aprepitant on lipid metabolism; to evaluate the effects of aprepitant on circulating proinflammatory cytokines and chemokines		
(see ref 33 for details)			

cART = combination antiretroviral therapy, HIV = human immunodeficiency virus, SP = substance P.

This study aimed to address the hypothesis that targeting the SP – neurokinin-1 receptor signaling pathway will affect tryptophan metabolism in vivo and thus may offer new therapeutic approaches for the treatment of HIV comorbidities such as chronic inflammation, associated with alterations in tryptophan metabolism.

## 2 Methods

### 2.1 Human samples

Archival samples from the phase 1B clinical trial “Anti-HIV Neuroimmunomodulatory Therapy with Neurokinin-1 Antagonist Aprepitant” were used (detailed^[34–36]^). Samples from 57 HIV-infected individuals (26 cART naïve individuals receiving aprepitant, 19 cART naïve individuals receiving placebo, and 12 individuals on a ritonavir-containing cART regimen receiving aprepitant) were utilized in this study. Placebo groups, when available, were matched based on gender, age, race, and viral loads to the treatment groups.^[35,36]^ Original studies were performed in outpatient settings. No dietary restrictions were imposed. The original studies were conducted at the AIDS Clinical Trials Unit and the Clinical and Translational Research Center (CTRC) of the Hospital of the University of Pennsylvania in Philadelphia, Pennsylvania, USA. All patients signed written informed consent. The study was sponsored by the National Institutes of Mental Health (NIH U19MH086336 and NIH U01MH090325 to SDD), approved by the IRB of the University of Pennsylvania and the US Food and Drug Administration (IND#75,558), and registered in Clinical Trials.gov (NCT00428519, NCT01300988, and NCT02154360).

### 2.2 Kynurenine and tryptophan enzyme-linked immunosorbent assay

Plasma concentrations of kynurenine (Rocky Mountain Diagnostics, Colorado Springs, CO, USA), tryptophan were quantified by enzyme-linked immunosorbent assay (ELISA) (both from Rocky Mountain Diagnostics, Colorado Springs, CO, USA), according to the manufacturer's recommendations. KTR was calculated as [plasma kynurenine (ng/mL)]/[plasma tryptophan (μg/mL)].

### 2.3 Statistical analyses

The results are expressed as the mean ± SD for replicate observations as indicated in the figure legends. Descriptive statistics, normality tests and paired *t* tests or Wilcoxon matched-pairs signed-rank tests (GraphPad Prism 8, GraphPad Software, LLC) were used to evaluate the significance of differences between parameters; *P* < 0.05 was considered statistically significant.

## 3 Results and discussion

**Figure 1:**
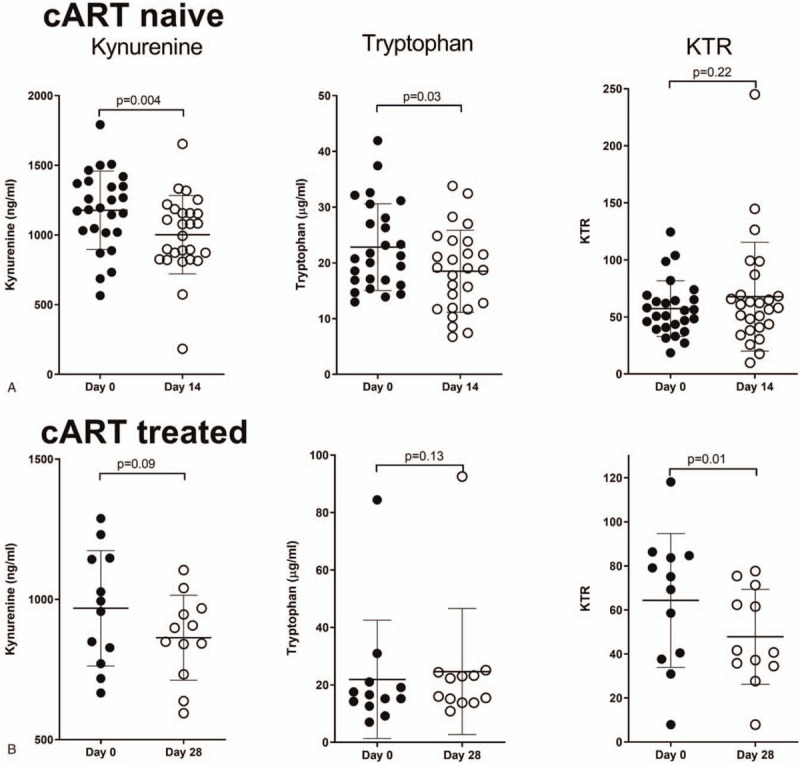
Aprepitant treatment has different effects on tryptophan catabolism in cART treated and cART naïve HIV-infected individuals. KTR was measured in plasma samples using kynurenine and tryptophan ELISAs. Kynurenine (ng/mL), tryptophan (μg/mL), and KTR in the plasma of (A). cART naïve (n = 26) and (B). cART treated (n = 12) HIV-positive individuals that were receiving aprepitant. Filled circles indicate values before and open circles indicate values after aprepitant treatment. Data are shown as mean ± SD. Significance was determined by two-tailed paired *t* test or Wilcoxon test, *P* < 0.05 was considered significant. cART = combination antiretroviral therapy, ELISA = enzyme-linked immunosorbent assay, HIV = human immunodeficiency virus, KTR = kynurenine to tryptophan ratio.

We determined the effect of aprepitant treatment on tryptophan metabolism using archival samples from HIV-infected individuals enrolled in clinical trials.^[34–36]^ Kynurenine and tryptophan plasma levels, in HIV-infected cART treated and cART naïve individuals, were affected differently by aprepitant treatment. In cART treated individuals aprepitant administration decreased kynurenine levels modestly, although not significantly, and increased tryptophan levels, resulting in an overall significant KTR decrease (mean of differences: −16.51 +/−18.19; paired *t* test: *P* = 0.01). However, in cART naïve subjects aprepitant administration decreased kynurenine and tryptophan levels, resulting in non-significant changes in overall KTR (Fig. 1). Among the 19 HIV infected individuals enrolled in the placebo group there was a small decrease in plasma tryptophan levels (mean of differences: −3.65+/−6.75; paired *t* test: *P* = 0.03) and no changes in the plasma levels of kynurenine (paired *t* test: *P* = 0.08) nor KTR (Wilcoxon test: *P* = 0.21) (data not shown).

Our results support the importance of tryptophan catabolism in the pathophysiology of chronic inflammation during HIV infection. Aprepitant treatment is associated with decreased levels of inflammation markers, including sCD163, IL-6, and SP, albeit with differences between cART treated and naïve populations; decreases in PD-1 expression on T-cells and sCD163 were observed only in cART naïve individuals.^[34,35]^ The differences between our 2 studies, in cART treated and cART naïve individuals, may be related to differences in initial baseline levels of pro-inflammatory markers (eg, the initial baseline of sCD163 in the HIV viremic population was in the 1500–4000 ng/mL range, while patients with undetectable viral loads had sCD163 levels in the 400–1800 ng/mL range) and this could affect tryptophan catabolism. The more profound effect of aprepitant in individuals on cART may be due to higher aprepitant plasma levels achieved with ritonavir-containing regiments. Aprepitant administration in combination with the CYP 3A4 inhibitor, ritonavir, resulted in a significant boost of aprepitant plasma levels. The mean peak aprepitant plasma concentrations on day 14 were 30.7 ± 15.3 μg/mL in the cART treated group vs 7.6 ± 3.1 μg/mL in the cART naïve patients.^[34,35]^

The specific immune mechanisms, by which stress and depression influence immunity and HIV disease progression and mortality, are not fully understood. Tryptophan depletion, or alternatively, increased IDO activity may affect the biosynthesis of the neuromodulators melatonin and serotonin, which may affect sleep and behavior, respectively. Many pathophysiological conditions including HIV, stress, and depression are linked to the release of extracellular ATP, which activates the inflammasome and results in the release of IL-1β and activation of TNFα, while inflammation-mediated immune activation can increase IDO activity leading to tryptophan depletion.^[37]^

Aprepitant and other neurokinin-1 receptor antagonists have immune-stimulatory and anti-inflammatory properties and studies suggest that they improve general well-being, reduce depression and promote sleep.^[38,39]^ In our cART treated cohort only 2 out of the 12 participants exhibited depressive symptoms (The Hamilton-17 Depression Rating Scale (HAM-D-17), the Hamilton Anxiety Scale (HAM-A)) and sleep disturbances at enrollment. Aprepitant treatment was associated with decreases in the HAM-D-17, HAM-A, and the Pittsburgh Sleep Quality Index in both individuals, detailed in Spitsin et al.^[34]^ This result may suggest that aprepitant's mode of action “protects” the neuromodulators melatonin and serotonin but the limited effect that we observed in this study does not allow for a definite conclusion. Further studies are required to demonstrate that this is the case. No other changes in overall symptoms or overall well-being were reported by the subjects during aprepitant treatment.

### 3.1 Study limitations

Our main conclusions are limited by the design of the original studies because the primary study objectives were focused on the safety and tolerability of aprepitant in HIV-infected individuals during the escalation of dose and treatment duration. As a result of the original study design, higher plasma concentrations of aprepitant were achieved in patients on cART. Based on the original study design there was also no placebo group associated with the cART treated patient population. However, since all of the aprepitant treated patients in the cART group were on stable therapy with complete viral suppression, we feel that there is minimal likelihood that the observed changes in KTR are due to factors other than aprepitant treatment, for example, due to the natural course of HIV infection. Tryptophan metabolism may be affected by many factors such as sex and diet. In our study, placebo groups, when available, were matched based on gender, age, race, and viral loads to the treatment groups, however, no dietary restrictions were imposed during the studies. Aprepitant administration affected metabolism by activating the CYP 3A4 enzyme in the liver. However, there is no evidence that aprepitant may activate directly other enzymatic pathways involved in tryptophan metabolism, such as IDO.

## 4 Conclusion

Our results support the hypothesis that targeting the SP – neurokinin-1 receptor signaling pathway offers new therapeutic approaches for the treatment of HIV comorbidities such as chronic inflammation, associated with alterations in tryptophan metabolism. Optimal conditions for such interventions, including dose and treatment duration, remain to be determined.

## Author contributions

**Conceptualization:** Sergei Spitsin, Jay Rappaport, Steven D. Douglas, Dwight L. Evans.

**Data curation:** Sergei Spitsin, Vasiliki Pappa.

**Formal analysis:** Sergei Spitsin, Dwight L. Evans, Jay Rappaport, Vasiliki Pappa, Annemarie Kinder, Steven D. Douglas.

**Funding acquisition:** Steven D. Douglas.

**Investigation:** Sergei Spitsin, Annemarie Kinder, Vasiliki Pappa.

**Supervision:** Sergei Spitsin, Dwight L. Evans, Steven D. Douglas.

**Writing – original draft:** Sergei Spitsin, Vasiliki Pappa, Dwight L. Evans, Jay Rappaport, Steven D. Douglas.

**Writing – review & editing:** Sergei Spitsin, Vasiliki Pappa, Dwight L. Evans, Steven D. Douglas.
